# Application of the *Gadidae* Fish Processing Waste for Food Grade Gelatin Production

**DOI:** 10.3390/md19080455

**Published:** 2021-08-09

**Authors:** Nikita Yu. Zarubin, Elena N. Kharenko, Olga V. Bredikhina, Leonid O. Arkhipov, Konstantin V. Zolotarev, Anton N. Mikhailov, Valeriya I. Nakhod, Marina V. Mikhailova

**Affiliations:** 1 Department of Technical Regulation, Russian Federal Research Institute of Fisheries and Oceanography, 17 Verkhnyaya Krasnoselskaya str., 107140 Moscow, Russia; zar.nickita@yandex.ru (N.Y.Z.); harenko@vniro.ru (E.N.K.); bredihinaov@rambler.ru (O.V.B.); arhipidis@gmail.com (L.O.A.); 2 Laboratory of Environmental Biotechnology, Institute of Biomedical Chemistry, 10 Pogodiskaya str., 119121 Moscow, Russia; myhas84@mail.ru (A.N.M.); kardavaleriya@yandex.ru (V.I.N.); m_mikhailova@mail.ru (M.V.M.)

**Keywords:** fish processing waste, Alaska pollock, Pacific cod, fish gelatin, gelling component

## Abstract

Waste from fish cutting (heads, swim bladders, fins, skin, and bones) is a high-value technological raw material for obtaining substances and products with a wide range of properties. The possibility of using waste from cutting fish of the *Gadidae* family: the Alaska pollock (*Gadus chalcogrammus*) and the Pacific cod (*Gadus macrocephalus*), processed in the coastal zone, is scientifically substantiated. In this work, a technology has been developed for processing accumulated waste from fish cutting in order to obtain fish gelatin, which is characterized by high protein content (more than 80.0%) and a full set of essential and nonessential amino acids. We studied the quality of fish gelatin obtained from wastes from cutting the fish of the *Gadidae* family. The possibility of using fish gelatin as a component of fish products is shown; the dose of its introduction into the fish products is substantiated. The data obtained made it possible to recommend the use of fish processing waste products as a gelling component and a source of amino acids in multicomponent food systems.

## 1. Introduction

Fish processing industry produces large amounts of by-products (heads, bones, fins, skin, etc.) worldwide. Some of those products are nutritionally valuable, so it is reasonable to evaluate the applicability of various fish by-products for different purposes, including human nutrition [1]. Fish collagen and fish gelatin (partially hydrolyzed collagen) can be derived from heads (initial collagen content: 9.2–33.0%), fins (initial collagen content: 0.8–8.0%), skin (initial collagen content: 2.0–12.6%), scales (initial collagen content: 0.8–6.0%), bones (initial collagen content: 9.0–19.0%), and dead fish bodies [2]. Fish collagen can be used for various purposes, including wound healing, tissue engineering, drug delivery, cell differentiation in cell culture, and skin care, or as a source of amino acids [3].

Gelatin is used as a gelling component for cooking. Mammalian gelatin is derived from porcine and bovine by-products, so there are some religious restrictions in its application [4]. Fish gelatin is a potential alternative to the mammalian gelatin for application in such cases. Besides that, fish gelatin can be produced for domestic use in local coastal communities earning their lives with fishing, so fish gelatin may help relieve local malnutrition there [5].

Some of the *Gadidae* fish species are among the most captured ones. The Alaska pollock was the second most captured in 2018 (3.4 million tonnes) [6]. The Pacific cod has been one of the most captured fish species in Russia for the last several years [7,8]. Generally, only 30–50% of the fish wet weight is used in the production of fish fillets [1]. Thus, it is reasonable to evaluate the applicability of the Alaska pollock and the Pacific cod by-products for food grade gelatin production. Besides that, if the mixture of the by-products is applicable and includes separated components, it will increase the economic valuability of the whole process because a tedious stage of sorting would not be necessary. Unfortunately, fish gelatin is applicable only for fish meals cooking due to fish odor [4]. Fish gelatin may be used for fish jelly or fish heated-mince production, so it is necessary to study the chemical composition of gelatin samples and some physicochemical properties of a gel prepared using gelatin (in comparison with a mammalian gelatin). In addition, it is necessary to study the amino acid content of gelatin samples for their nutritional value estimation (in comparison with a mammalian gelatin). If gelatin and the products prepared with it have sufficient quality parameters and nutritional value, the possibility of their application will be substantiated and this way of the *Gadidae* fish processing waste utilization will be an option to be considered.

## 2. Results

### 2.1. Chemical Analysis of By-Products

All the types of by-products of both fish species were analyzed for total protein, total fat, collagen, ash, and water contents (Table 1).

According to the data in Table 1, it can be concluded that the chemical parameters do not have strong differences. All types of waste were high in total protein content (over 15%) and collagen content (over 10%) in contrast to the total fat and ash contents. In this regard, the mixing of waste will not lead to a deterioration in the chemical composition, namely the total protein and collagen contents, which was confirmed by mixture sample parameters (ANOVA; *p* < 0.1 for each). The collagen content was about 80% of the total protein in the mixture sample, which is positive for obtaining fish gelatin.

Thus, in further work, we used a mixture of by-products from pollock and cod in equal proportions, which is reasonable, since these fish species belong to the same family and have a similar chemical composition. In addition, pollock and cod are most widely used at fish processing plants in Russia for the production of fish products of various types, which implies their complete cutting to fillets, with subsequent waste collection [9].

### 2.2. Fish Gelatin Quality Parameters Assessment

Fish gelatin samples were prepared from the mixtures of by-products; their quality parameters were compared with the parameters of the K-10 standardized mammalian gelatin derived from porcine and bovine by-products according to [10] (Table 2).

According to the data in Table 2, it can be concluded that fish gelatin met the standardized requirements. Differences in color and odor of the product were observed due to the organoleptic properties of the fish by-products. The fish gelatin solution was more transparent in comparison with the K-10 gelatin solution. The melting point of the jelly was lower than the standardized value, which was explained by the fact that fish collagen is sensitive to heating due to unstable cross-links compared to mammalian collagen. The dried fish gelatin contained a significant amount of protein (up to 92.6%), where collagen accounted for approximately 74.1% of total protein, and therefore it can be recommended for use as an independent food supplement (a source of collagen proteins) and also as a structuring agent.

### 2.3. Fish Gelatin Amino Acid Analysis

The amino acid analysis of the fish gelatin was performed (Table 3).

The amino acid composition of fish gelatin (Table 3) was characterized by the presence of proline and hydroxyproline (9.76% and 8.51%, respectively), which is a feature of the connective tissue of fish. At the same time, the content of glycine was high (33.2%). Glycine, proline, and hydroxyproline are the most important amino acids in collagen, accounting for 50% of the total amino acids in the protein. The content of proline and hydroxyproline is especially important for the gelling effect and stabilization of the collagen triple helix due to its ability to bind hydrogen through the OH group, which also affects the technological properties of fish gelatin, such as gel-forming ability and strength of a gel prepared using it.

### 2.4. Fish Gelatin Molecular Weight Analysis

The result of the analysis indicated that the fish gelatin consisted of fragments of collagen fibers (102.2 kDa on average) formed during enzymatic hydrolysis, which contributed to the formation of dense jelly gels.

### 2.5. Assessment of Quality Parameters of Fish Heated-Mince Prepared Using Fish Gelatin

Fish heated-mince samples without gelatin (control) and with added 2%, 4%, 6%, 8%, and 10% gelatin were prepared. Some quality parameters of the samples were estimated (Table 4).

Alaska pollock fillets were used as a core component for the preparation of fish heated-mince samples, because they have insufficient functional, technological, and structural quality values. The introduction of fish gelatin could potentially improve these properties and structure of fish heated-mince.

One of the most important indicators of heated-mince is the moisture absorption capacity (MAC). During heat treatment, physicochemical and colloidal changes occur; although some water and fat from raw fish mince are lost, some water is held in the mince, which can be characterized by water-holding capacity (WHC). At the same time, WHC characterizes the amount of water separated during the heat treatment. This parameter is associated with the output of finished products [11].

The analysis of the MAC dynamics showed that it grew with gelatin content increase. The highest MAC value was reached at a 10% dosage of fish gelatin (ANOVA; *p* < 0.1). A similar dynamic for WHC was observed, but the sample with 8% gelatin had a maximum WHC value (ANOVA; *p* < 0.1). It is explained by the clasterization and gelatinization of the gelatin components during heat treatment.

The ultimate shear stress (USS) value gradually increased with an increase in the dose of fish gelatin and reached a maximum at a 10% dosage, where fish heated-mince had the densest structure. It is explained by an increase in collagen content in the fish heated-mince, which led to an improvement in its hydrophilic properties. As a result of the presence of dispersed collagen fibers, water absorption was enhanced and USS value increased. Thus, the presence of fish gelatin promotes structure formation and an increase in the density of fish heated-mince.

Fat-absorption capacity (FAC) dynamics with an increase in the content of fish gelatin was similar to the dynamics of WHC.

### 2.6. Sensory Evaluation of Fish Heated-Mince Prepared Using Fish Gelatin

**Figure 1:**
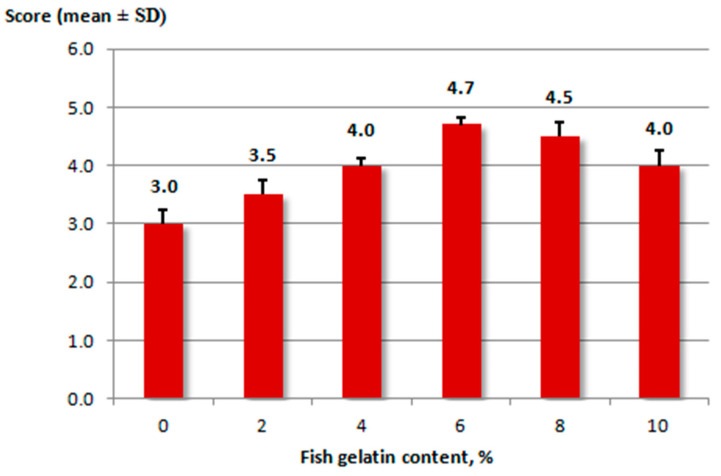
Results of the sensory evaluation of fish heated-mince with or without fish gelatin.

Fish heated-mince samples without gelatin (control) and with added 2%, 4%, 6%, 8%, and 10% gelatin were evaluated (Figure 1).

All the samples had a gray color, moderately salty and pleasant aroma, with the taste of the finished product, typical of the fish used. The samples with added 6% and 8% gelatin had the highest score on a 5-point scale (ANOVA; *p* < 0.1) (Figure 1). The control sample (0% gelatin) had a looser consistency. Samples with added 6% and 8% fish gelatin had soft and juicy consistency. The sample with added 10% fish gelatin was rated 4 points; this one had a lighter color and a firmer consistency, which increased its rigidity compared to the other samples.

## 3. Discussion

As a result of this study, the possibility of solving the problems of processing waste from cutting fish of the *Gadidae* family was substantiated. The approach makes it possible to produce fish gelatin with high protein content (>90%) and quality parameters not inferior to those of food grade gelatin derived from porcine and bovine by-products. Differences with mammalian gelatin in smell, melting point, and solubility were noted; they are associated with the organoleptic characteristics of raw fish-collagen-containing materials and the structure of collagen.

Collagen and gelatin derived from cold-water fish by-products have a lower hydroxyproline content and a lower thermal stability than collagen and gelatin from warm-water fish by-products. This phenomenon is caused by the involvement of hydroxyproline in the formation of a chain of hydrogen bonds that stabilizes the triple helical structure of collagen [11,12,13,14,15,16]. At the same time, the technological features of obtaining fish gelatin also affect the melting point. Due to the lower contents of proline and hydroxyproline, which are involved in the stabilization of the triple superstructure of collagen, the gelation process is slower and the fish gelatin-based gel melts at a lower temperature, in contrast to the gel with gelatin derived from another type of raw material. It should be noted that fish gelatin dissolves faster than gelatin derived from porcine and bovine by-products, because fish collagen has lower molecular fractions [12,17].

The fish gelatin derived from *Gadidae* fish processing by-products contains a full set of essential and nonessential amino acids. The amino acid composition of the gelatin derived from a mixture of the Alaska pollock and the Pacific cod was close to the previously published composition of the Alaska pollock skin [18] and the Atlantic cod skin [19]. Thus, our fish gelatin can be recommended for use as an independent food supplement (a source of amino acids). The amino acid composition of our fish gelatin is quite similar to that of porcine skin gelatin (ANOVA; *p* < 0.1), which is also characterized by high contents of glycine, proline, and hydroxyproline [20].

The cost of producing gelatin from porcine and bovine bone raw materials in Russia is about US$2350–2650 per ton of product, including the cost of raw materials, equipment, and labor [21]. The cost of fish gelatin is lower and ranges from US$1200 to US$1300 per ton of product, which is achieved due to the lower cost of raw materials and a more simplified production line.

The possibility of using fish gelatin in the production of fish culinary products based on fish heated-mince has been studied. According to the results of this study, the recommended content of fish gelatin in the fish heated-mince is 6–8%. The appropriate samples showed high values of MAC, WHC, FAC, and USS, which is a positive factor in the formation of the consistency of fish products. A higher dosage of fish gelatin is not recommended, since in this case, despite the high functional and technological properties, there is deterioration in the organoleptic characteristics of the finished product.

Thus, the fish gelatin derived from the *Gadidae* fish processing waste can be rationally a natural gelling component and an amino acid source. The fish gelatin being studied meets the standardized quality requirements of food grade mammalian gelatin and has a quite similar amino acid composition. The acceptable cost of the *Gadidae* fish gelatin production makes it possible to use, instead of mammalian gelatin, especially in coastal communities and/or in cases of religious restrictions of mammalian gelatin use. Besides that, the use of fish gelatin, instead of mammalian gelatin, would help to utilize the *Gadidae* fish processing waste.

## 4. Materials and Methods

### 4.1. Samples of By-Products

The frozen fish samples of the Gadidae family (the Alaska pollock (*Gadus chalcogrammus*) and the Pacific cod (*Gadus macrocephalus*)) were obtained from local fish dealers in Khabarovsk region, Russia. The fish were caught by fishing companies in the northern part of the Sea of Okhotsk. The adult healthy-looking fish were chosen for the study. The mixture for gelatin production was prepared from fish by-products in equal proportions by weight: skin with scales + tails and fins + spinal bones + viscera (without liver), with each by-product from both species in equal proportion. Five samples of the mixture were prepared for comparison.

### 4.2. Total Protein Content Determination

Total protein content was determined using the Kjeldahl method with a Kjeltec 1002 System Distilling Unit (Tecator AB, Höganäs, Sweden); total protein content was calculated as 6.25 × total N content. Each measurement was made in triplicate.

### 4.3. Total Fat Content Determination

A sample (5–10 g) was ground in a mortar with Na_2_SO_4_ (sample-to-salt weight ratio: 1:3) for dehydration. Next, the dehydrated sample was placed into a boiling flask of a Soxhlet extractor, and diethyl ether was added; the ether volume was about 1.5 times the flask volume. Next, the condenser was adjusted, cooling water was let through, and the flask was gently heated in a water bath for 12 h.

After extraction, the ether was distilled off, and the fat collected in a small preliminarily weighed flask was dried at 105 °C to constant weight and then weighed. Each measurement was made in triplicate.

### 4.4. Ash Content Determination

A sample (about 4 g) was digested in a muffle furnace at 500–700 °C for about 1 h to constant weight; the ash was collected and weighed. Each measurement was made in triplicate.

### 4.5. Water Content Determination

The water content (moisture) of a sample was approximately estimated as: 100% − total protein content (%) − total fat content (%) − ash content (%).

### 4.6. Collagen Content Determination

A homogenized sample (about 50 mg) was hydrolyzed by autoclaving with 1.0 mL of 6 M HCl in sealed tubes at 3.5 bar pressure for 3 h. One milliliter of 0.01 M CuSO_4_, 1 mL of 2.5 M NaOH, and 1 mL of 6% H_2_O_2_ was added into the sample tube and a blank tube with 1 mL of distilled water. The solutions were mixed and shaken occasionally during a period of 5 min and placed in a water bath at 80 °C for 5 min with frequent intensive shaking. Next, the tubes were cooled in an ice water bath, and 4 mL of 3.0 N (1.5 M) H_2_SO_4_ was added with shaking. Then, 2 mL of p-dimethylaminobenzaldehyde solution in n-propanol were added with thorough shaking. The tubes were heated at 70 °C for 16 min and cooled in tap water. The absorption at 540 nm of the prepared solutions was measured with a PE-5300VI spectrophotometer (Ecroskhim, St. Petersburg, Russia). Hydroxyproline content was calculated using a previously made calibration with hydroxyproline standard (Sigma-Aldrich, St. Louis, MO, USA). Collagen content was calculated as 7.4 × hydroxyproline content [22]. Each measurement was made in triplicate.

### 4.7. Fish Gelatin Preparation

The samples of by-products were preliminarily three times treated with a 10% aqueous solution of citric acid for 60 min with constant stirring. After washing, the samples were frozen at −25 °C for 2 h and later crushed. After crushing, the samples were preliminarily heated at 80–90 °C for 20–25 min in water (sample-to-water volume ratio: 1:2) and cooled to 35–40 °C. Next, the resulting mass was hydrolyzed with a mixture of enzyme preparations “Food collagenase” (Bioprogress, Shchyolkovo, Russia) and “Protepsin” (Endocrine Enzymes Plant, Moscow, Russia) at 40 °C for 4 h; the enzyme-to-sample ratio was 1:1000 by weight. “Food collagenase” was produced from crab hepatopancreases and designed for collagen hydrolysis; “Protepsin” was a complex of animal-derived proteinases designed for raw meat processing. The application of a mixture of both preparations permitted to hydrolyze the proteins of both connective and muscle tissues. Next, the enzymes were inactivated by heating at 70 °C for 15 min, and the broth was decanted.

To reduce energy consumption for drying, the obtained gelatin broth was preliminarily thickened by ultrafiltration using the UF-401/402 pilot filtration system (BioTechno Group, Moscow, Russia) at 48–52 °C to obtain a dry matter content of 25–30%. The membranes of the ultra-permeable membrane (UPM) type made of aromatic polysulfonamide (Sulfon-4T 50M type, Vladipor, Vladimir, Russia) were used for ultrafiltration; the retention capacity of the membrane was 20–100 kDa.

The gelatin concentrate was dried in an SD-1000 spray dryer (EYELA, Japan) at 50–60 °C by spraying through 0.7 mm nozzles. The yield of dry fish gelatin powder was 18% ± 2%.

### 4.8. Odor and Flavor Estimation

Ten grams of gelatin were dissolved in 90 mL of distilled water in a plugged flask and heated at 40–50 °C for 1 h. Odor was estimated organoleptically after unplugging the flask. Next, the solution was cooled to 17–19 °C in an open glass. The flavor of the cooled solution was estimated organoleptically.

Odor and flavor were estimated as weak, moderate, strong odor/flavor, or none odor/flavor. The nature of the odor/flavor was also estimated.

### 4.9. Particle Size and Small Particle Fraction Determination

The determination of these parameters was based on sieve analysis with two parallel sieves (mesh size: 10 and 0.5 mm) with a receiver tray beneath. One hundred grams of gelatin was sifted through a sieve system with a shaking rate of 1 shake per second. The biggest gelatin particles remaining on the upper sieve were selected and measured manually with a caliper. The gelatin collected in a tray was weighed for the small particle fraction calculation. Each measurement was made in triplicate.

### 4.10. Dissolution Time Determination

Ten grams of gelatin were mixed with 100 mL of distilled water at 15–18 °C and left for swelling at this temperature for 30 min. Then, the mixture was thermostated at 40 °C, until the gelatin was fully dissolved. Dissolution time was measured, from the time when the temperature increased to 40 °C, by the mixture until its full dissolution. Each measurement was made in triplicate.

### 4.11. Preparation of Gelatin Solution and Jelly

A portion of gelatin (1.03 × calculated mass) was placed in a flask; the calculated amount of water was added; the mixture was gently stirred until its homogeneity was achieved, covered and kept at room temperature with periodical stirring for 1.5 h for gelatin swelling. The flask with the swollen gelatin was thermostated at 55 °C for 30–40 min with careful stirring for gelatin dissolution. Then, the solution was filtered through 4 layers of gauze and cooled until the temperature of 41–43 °C was realized. When a 10% gelatin solution was cooled to room temperature, it turned into jelly.

### 4.12. Determination of pH

A SevenExcellence pH meter with InLab Expert Go-5m-ISM electrode (Mettler Toledo, Greifensee, Switzerland) was used to determine the pH value of the gelatin solution. Each measurement was made in triplicate.

### 4.13. Viscosity Determination

The viscosity of the gelatin solution was determined using a VPJ-2 U-tube viscosimeter (Ecroskhim, St. Petersburg, Russia). A portion of gelatin (20 g dry weight; the actual weight was calculated using the results of water content determination) was placed in a flask, the calculated amount of water was added, the flask was closed with a rubber plug with a narrow hole (for an air outlet), and the solution was kept at room temperature with periodical stirring for 1 h for gelatin swelling. The flask with the swollen gelatin was thermostated at 65 °C for 30 min with careful stirring for gelatin dissolution. Then, the solution was cooled to 41–43 °C. The determination of viscosity was carried out no later than 30 min after cooling the solution. Each measurement was made in triplicate.

The solution was filtered through a glass filter, poured into a viscometer and thermostated at 4.0 °C for 10–15 min, and the outflow time was measured. Viscosity was calculated using the values of the outflow time and the viscometer constant.

### 4.14. Melting Point Determination

The melting point of the jelly was determined using the Cambon’s fusiometer consisting of a brass crucible and a brass rod with a hole for hanging. The rod was placed onto the center of the crucible bottom. Next, the crucible was filled to the top with a 10% gelatin solution.

The filled crucible was first kept for 30 min at room temperature and then at 11 °C for 1 h for gelling. Next, the crucible was placed in a glass of 20 °C water and the rod was hung up, so that the crucible edge was at the water surface level in the glass.

At the same time, a thermometer was attached at 0.5 cm from the crucible, deepening its ball to the level of the crucible bottom. The glass with the installed system was placed in a water bath and was uniformly heated, increasing the bath temperature by 1 °C for 3 min until the crucible was separated from the rod. The melting point of gelatin-based jelly was the temperature of the water, at which the crucible was separated from the rod and fell down to the glass bottom. Each measurement was made in triplicate.

### 4.15. Transparency Determination

The 5% solution was prepared and cooled to 40 °C. Its transparency was measured as optical transmission using a KFK-3-01 colorimeter with a blue filter (Zagorsk Optical-Mechanical Plant, Sergiyev Posad, Russia). Distilled water was the blank sample. Each measurement was made in triplicate.

### 4.16. Gel Strength Determination

Gel strength was determined with a VTs-1 Valent’s device (PuAZ, Odessa, Ukraine). Fifty milliliters of the gelatin solution were poured into the device vessel, and then, the vessel was cooled at room temperature for gelling and later cooled to 8 °C for 18 h. Next, the vessel was warmed to reach a temperature of 15 °C in a cool water bath for 2 h, and the gel strength was immediately measured. The load mass was increased at a rate of 10–12 g per second, until the jelly was crushed. The load mass at the crushing time was considered as gel strength. Each measurement was made in triplicate.

### 4.17. Impurities Content Determination

One liter of 10% gelatin solution was prepared and filtered through a sieve. Next, the sieve was washed with hot water (65 °C) for 5 min. The sediment was collected, dried to constant weight and then weighed. Each measurement was made in triplicate.

### 4.18. Amino Acid Analysis

Ten milligrams of the sample were dissolved in 1 mL of distilled water. The solution was 25 times diluted, and 50 μL of the solution were dried up in an ampoule. Then, 100 μL of 6 M HCl were added, and the ampule was sealed under vacuum. Acidic hydrolysis was performed at 110 °C for 24 h. After that, the ampoule was opened, and the solution was dried up in the Eppendorf 5301 vacuum concentrator (Eppendorf, Hamburg, Germany). Finally, 50 μL of 0.1 M HCl were added to the dried sediment.

The amino acid analysis was performed using Agilent 1200 series chromatographic system equipped with a fluorescent detector and ZORBAX Eclipse AAA (5 μm; 4.6 mm × 150 mm) column (Agilent Technologies, Santa Clara, CA, USA). The mobile phases were a 40 mM phosphate buffer solution with pH 7.8 (solution A) and 80% water solution of acetonitrile (solution B). Borate buffer with pH 10.2 and *o*-phtalaldehyde were used for amino acid derivatization. Each measurement was made in triplicate. See [23] for details.

### 4.19. Molecular Weight Analysis

The average molecular weight of gelatin components was estimated using the viscometric method with a VPJ-2 U-tube viscosimeter (Ecroskhim, St. Petersburg, Russia). The solution from the analysis by Kjeldahl method (see Section 4.2) was used for this procedure. A set of four serially diluted solutions was made from that one. The viscosity of all those solutions and 1.0 M KCl as a standard was determined as described above (Section 4.13). The value of intrinsic viscosity was determined graphically as:

(1) $$\left[\eta \right]=\underset{c\to 0}{\lim}\frac{1}{c}\ln\frac{\eta }{\eta_{0}},$$

where *c* is calculated as 100/(total N content in a solution) in g/mL;

*η* and *η*_0_ is the viscosity values of a solution and 1.0 M KCl, respectively.

The average molecular weight (in Da) was calculated using the Mark–Houwink equation:

(2) $$M={\left(\frac{\left[\eta \right]}{k}\right)}^{1/a},$$

where *k* and *a* are constants for each type of biopolymer. For gelatin at room temperature, *k* = 0.1681 mL/g and *a* = 0.9211 [24].

Each measurement was made in triplicate.

### 4.20. Preparation of Fish Heated-Mince Samples

Alaska pollock fillets were minced with a meat grinder; NaCl was added to the mince (1% of mince weight), and it was mixed. Fish gelatin was added in an appropriate amount (0%, 2%, 4%, 6%, 8%, or 10% of mince weight): it was diluted in water (fish gelatin-to-water volume ratio: 1:3) and heated to 67 °C in a water bath, and then gelatin solution was incorporated into the mince. The mince was mixed and kept for 30 min for stabilization. MAC and USS were determined before heat treatment.

For heat treatment, the samples were placed into 250 mL glass jars with hermetically sealed lids, the lids were sealed and the samples were heated at 80–85 °C for 85 min. WHC and FAC were determined after heat treatment.

### 4.21. MAC Determination

MAC was determined before heat treatment. A preliminarily weighed portion of a heated-mince sample was placed onto a piece of filter paper and gently pressed with a glass stick, until water release ended. The area of the water stain was measured, and the volume of water was calculated using a preliminarily made calibration. MAC was calculated as a ratio of water volume to heated-mince portion. Each measurement was made in triplicate.

### 4.22. USS Determination

USS was determined before heat treatment. USS was measured using the APN-360MG4 automatic penetrometer (SKB Stroypribor, Chelyabinsk, Russia). A portion of a heated-mince sample was put into a glass. The time of cone hold after crush was set (5 s), the sample was lifted until it touched the cone, and the measurement procedure was initiated. The USS value was calculated (in Pa) using the Rehbinder equation:

(3) $$\theta_{0}=\frac{km}{h^{2}},$$

where *k* of 2.1 N/kg is a Rehbinder’s constant for the cone used with a 60° apex angle;

*m* of 0.05069 kg is the cone + holding bar weight;

*h* is the cone immersion depth being measured (in meters).

Each measurement was made in triplicate.

### 4.23. WHC Determination

WHC was determined after heat treatment. A preliminarily weighed portion of a heated-mince sample (30–250 g) was placed into a preliminarily weighed glass jar with a lid, the lid was hermetically sealed, and the sample was heated until the temperature in the core of the portion reached 70 °C. Next, the sample was cooled, the broth was decanted, and the glass with the sediment was weighed. WHC was calculated as a relative loss of the sample weight. Each measurement was made in triplicate.

### 4.24. FAC Determination

FAC was determined after heat treatment. A preliminarily weighed portion of a heated-mince sample (30–250 g) was placed into a glass jar with a lid. Vegetable oil (30% of the sample weight) was added to the jar, the lid was hermetically sealed, and the mixture was heated until the temperature in the core of the portion reached 70 °C. Next, the mixture was cooled, and the broth was decanted. The broth was heated at 60 °C for 20 min in a water bath. The fat fraction of the broth was collected and weighed. FAC was calculated as: (fat fraction weight − initial oil weight)/heated-mince sample portion weight. Each measurement was made in triplicate.

### 4.25. Sensory Evaluation

The fish heated-mince samples were evaluated organoleptically by overall appearance, color, odor, flavor, and consistency with 10 adult people. Each person gave an integer score on a 5-point scale (from 1 to 5: 1 point for an uneatable sample; 2 points for an eatable but unsatisfactory sample; 3 points for a satisfactory sample which needs to be improved; 4 points for a satisfactory sample which can be slightly improved; 5 points for a perfect sample) to each heated-mince sample and described his/her impressions verbally. Each evaluation was made in triplicate.

## 5. Conclusions

The gelatin derived from the *Gadidae* fish (the Alaska pollock and the Pacific cod) processing waste can be rationally a natural gelling component and an amino acid source. The fish gelatin being studied meets the standardized quality requirements of food grade gelatin. It contains a full set of essential and nonessential amino acids and has an amino acid composition quite similar to that of mammalian gelatin.

The *Gadidae* fish gelatin can be used in the production of fish culinary products based on fish heated-mince. The optimal content of gelatin is 6–8%, according to the determined heated-mince quality parameters and the results of sensory evaluation.

The acceptable cost of *Gadidae* fish gelatin production makes it possible to use, instead of mammalian gelatin, especially in coastal communities and/or in cases of religious restrictions of mammalian gelatin use.

The use of *Gadidae* fish gelatin, instead of mammalian gelatin, would help to utilize the *Gadidae* fish processing waste.

## Figures and Tables

**Table 1:** Chemical parameters of by-products of the Alaska pollock and the Pacific cod. Content values are expressed as % by wet weight (mean ± SD; *n* = 5).

By-Product Type	Water Content		Total Protein Content		Collagen Content		Total Fat Content		Ash Content	
	Pollock	Cod	Pollock	Cod	Pollock	Cod	Pollock	Cod	Pollock	Cod
Heads	78.55 ± 1.44	78.21 ± 1.43	16.41 ± 0.40	15.71 ± 0.39	11.37 ± 0.28	10.02 ± 0.24	1.34 ± 0.03	0.77 ± 0.01	3.70 ± 0.09	5.37 ± 0.14
Skin + scales	76.32 ± 1.41	75.79 ± 1.39	19.49 ± 0.48	18.61 ± 0.46	15.23 ± 0.38	14.38 ± 0.34	1.23 ± 0.03	1.03 ± 0.02	2.97 ± 0.06	3.56 ± 0.08
Tails and fins	77.67 ± 1.42	79.55 ± 1.45	16.71 ± 0.41	14.38 ± 0.36	13.56 ± 0.33	11.52 ± 0.27	1.28 ± 0.03	0.53 ± 0.01	4.37 ± 0.12	5.54 ± 0.14
Spinal bones	79.34 ± 1.46	74.32 ± 1.36	15.68 ± 0.38	18.25 ± 0.45	11.21 ± 0.28	13.51 ± 0.32	0.88 ± 0.01	0.75 ± 0.01	4.10 ± 0.12	6.68 ± 0.15
Viscera (without liver)	75.32 ± 1.38	74.47 ± 1.36	19.10 ± 0.47	19.96 ± 1.36	15.31 ± 0.38	14.98 ± 0.35	2.35 ± 0.06	2.58 ± 0.06	3.23 ± 0.08	2.99 ± 0.06
Mixture sample	74.41 ± 1.36		21.23 ± 0.52		17.07 ± 0.42		1.31 ± 0.03		3.05 ± 0.10	

**Table 2:** Quality parameters of fish gelatin derived from a mixture of fish by-products (the Alaska pollock + the Pacific cod) (mean ± SD; *n* = 5) in comparison with those of a standardized mammalian gelatin.

Parameter	Fish Gelatin	K-10 Mammalian Gelatin Standardized by [10]
Appearance	Powder	Granules, grains, plates, and powder
Color	Smoky white	From light yellow to yellow
Odor	Fishy (weak)	None
Flavor	Insipid	Insipid
Particle size, mm	3 ± 0.2	≤5
Small particle fraction, %	None found	≤30
Dissolution time, min	18 ± 2	≤25
pH of a 1% solution	5.4 ± 0.3	5–7
Viscosity of a 10% solution, mPa·s	38.5 ± 1.4	≥18.5
Melting point of jelly with 10% of gelatin, °C	26 ± 1	≥30
Transparency of a 5% solution, %	74.5 ± 1.3	≥50
Gel strength of jelly with 10% of gelatin, g	1800 ± 120	≥1000
Water content, %	5.8 ± 0.4	≤16
Ash content, %	1.6 ± 0.2	≤2
Total protein content	91.7 ± 0.9	Not standardized
Collagen fraction of total protein, %	73.2 ± 0.9	Not standardized
Total fat content, %	0.9 ± 0.15	Not standardized
Impurities content, %	None	None allowed

**Table 3:** Amino acid contents of fish gelatin derived from a mixture of fish by-products (the Alaska pollock + the Pacific cod) (mean ± SD; *n* = 5).

Amino Acid	Content, % of Total Protein
Ala	6.59 ± 0.61
Asp	7.29 ± 0.11
Arg	6.08 ± 0.14
Cys	0.10 ± 0.02
Glu	12.22 ± 3.23
Gly	33.20 ± 0.44
His	1.61 ± 0.07
Hyp	8.51 ± 0.36
Ile	2.57 ± 0.11
Leu	4.64 ± 0.41
Lys	5.66 ± 0.12
Met	0.03 ± 0.007
Phe	2.51 ± 0.13
Pro	9.79 ± 0.19
Ser	4.23 ± 0.21
Thr	2.53 ± 0.09
Tyr	0.03 ± 0.004
Val	2.94 ± 0.25

**Table 4:** Quality parameters of fish heated-mince samples with or without fish gelatin (mean ± SD; *n* = 5).

Parameter		Fish Gelatin Content, %					
		0	2	4	6	8	10
**Before heat treatment**	MAC ^1^, %	64.46 ± 1.05	68.34 ± 1.22	73.53 ± 1.44	79.48 ± 1.67	83.72 ± 1.78	84.23 ± 1.89
	USS ^2^, MPa	0.66 ± 0.02	0.84 ± 0.02	0.96 ± 0.02	1.34 ± 0.02	1.58 ± 0.02	1.67 ± 0.02
**After heat treatment**	WHC ^3^, %	71.34 ± 1.34	74.35 ± 1.51	76.81 ± 1.62	78.32 ± 1.66	79.65 ± 1.73	79.44 ± 1.67
	FAC ^4^, %	55.23 ± 0.71	57.83 ± 0.80	59.55 ± 0.88	61.23 ± 0.95	63.25 ± 0.99	62.97 ± 0.97

^1^ Moisture absorption capacity. ^2^ Ultimate shear stress. ^3^ Water-holding capacity. ^4^ Fat-absorption capacity.
